# Senile Cataract in Patients with Diabetes with and Without Diabetic Retinopathy: A Community-Based Comparative Study

**DOI:** 10.1007/s44197-021-00020-6

**Published:** 2021-12-07

**Authors:** Khalid Mohammad Alabdulwahhab

**Affiliations:** College of Medicine, Majmaah University, 4315, King Abdulaziz, Majmaah, 6514-15362 Kingdom of Saudi Arabia

**Keywords:** Type 2 diabetes, HbA1c, Insulin, Saudi Arabia, Hypertension, Risk factor, Age-of-onset

## Abstract

**Aim:**

We compare the incidence rates of cataract in persons with diabetes with and without diabetic retinopathy in Saudi Arabia, for the first time. In addition, we explored the role of new factor, diabetes age of onset and several other known factors.

**Methods:**

In a community-based cross-sectional study, 334 persons with diabetes type 2 were randomly selected from a diabetic register. Detailed history and comprehensive ophthalmic examination was done at an eye clinic. Body Mass Index, blood pressure and glycosylated hemoglobin were also recorded.

**Results:**

In 668 eyes, cataract and diabetic retinopathy were present in 35.5% and 32.2%, respectively. Diabetic retinopathy, age, duration of diabetes and systolic BP were found to be independent risk factors for cataract. Whereas, gender, BMI, HbA1c use of insulin and diastolic BP have no significant association with cataract. Persons with cataract had significantly higher age of onset of diabetes. Most of the cataracts were cortical followed by PSC, while minority were nuclear.

**Conclusion:**

DR is an independent risk factor of developing cataract in persons with diabetes. Others are age, duration of DM and hypertension. Age-of-onset of DM is a new factor we report it to be significantly associated with cataract.

## Introduction

Diabetes mellitus (DM) is one of the most common chronic systemic diseases and a major cause of visual loss worldwide [1–3]. According to the International Diabetes Federation, approximately 463 million adults aged 20–79 years were living with diabetes and by 2045 this is expected to rise to 700 million [4]. Among the top 10 countries in the world for diabetes prevalence in 2010, Saudi Arabia occupies the third place and is expected to continue to retain this position until 2030 [5]. The prevalence of DM in Saudi Arabia, according to the latest local study, was 30% [6].

DM can affect all ocular tissues; one of them is the human lens, which causes lens opacification (cataract). Globally, cataracts remain the 2nd-leading cause of blindness, affecting approximately 18 million people [7]. Cataract is a major cause of visual impairment in the general population [8, 9] as well as in the population with diabetes [10–12]. Further, a causal biochemical relationship between DM and cataract development has been proven in scientific research [13–15]. Clinical studies have revealed that DM is one of the well-known risk factors for cataract, and that cataracts occur 2–5 times more frequently in patients with DM than in those without DM [13, 16–19].

DM and cataracts, as independent diseases, are major health concerns and impose immense economic burden, especially in developing countries where DM and cataract treatment options are not optimal [20]. Furthermore, aging is a known risk factor for both diseases, which causes further burden on health care services [21]. To effectively prevent and treat blindness, it is important to identify the risk factors that play a role in the association between DM and cataract.

Despite extensive research on DM and cataract comorbidities, data from developing low- and middle-income countries are scarce [22]. Therefore, in this population-based study, we aimed to assess the incidence rates of cataract in patients with diabetic retinopathy (DR) and compare the data with individuals without DR from a population with diabetes in a developing country, Saudi Arabia. Furthermore, we explored the role of several other clinical factors and their association with cataract and cataract subtypes in patients with diabetes, such as age of onset, duration and control, blood pressure, and obesity.

Isolating such manageable factors, leading to the development of cataracts, will help health authorities build health programs that will lower the need for cataract surgery. Furthermore, it is especially important to reduce the demand for health resources in low-income countries.

## Subjects

### Study Design and Patients

This community-based, cross-sectional study aimed to determine the prevalence of senile cataract in patients with type 2 DM with DR compared to those without DR. The study subjects were residents of Majmaah, a medium-sized city in central Saudi Arabia with a population of approximately 60,000. The DM register is located at the eight primary health care centers (PHC) distributed throughout the city, which is continuously updated as new patients are diagnosed.

All patients with type 2 DM included in the diabetes registry (n = 1890 patients) were included in the study population. The sample was chosen from all PHCs with an equivalent proportion of the number of patients with type 2 DM in each PHC using a systematic random sampling technique. The chosen patients were given appointments at the eye clinic at the central hospital of the city for examination.

### Ethical Statement

The study was conducted from February 2020 to March 2021. This study was approved by the Ethical Review Committee of Majmaah University. Written informed consent from the patients was obtained before conducting the interviews and examinations.

### Materials and Methods

A detailed history, including data on demographics, past history of diabetes, and ocular problems was obtained from all patients. Height and weight were measured to calculate the body mass index (BMI). Blood pressure (BP) was measured using a blood pressure monitor (GE Dinamap). The patient was considered hypertensive if they reported hypertension in their medical history, or if their BP readings satisfied the World Health Organization definition of hypertension, (systolic of ≥ 140 or diastolic of ≥ 90) [23]. All patients underwent blood tests for glycosylated hemoglobin (HbA1c).

Best corrected visual acuity was measured using a Snellen distance vision screen by an optometrist, and then a senior ophthalmologist performed a comprehensive ophthalmic examination. The crystalline lens was assessed using a slit-lamp bio-microscope (Haag Streit, Germany). The cataract grade was determined according to the Lens Opacity Classification System (LOCS) III with direct reference to photographic standards at the slit lamp [24]. Patients with pseudophakic eyes were considered to have cataracts in the analysis. Pupils were dilated using 0.5% tropicamide and the posterior segment was evaluated using an indirect ophthalmoscope (Keeler, UK), as well as using a slit-lamp bio-microscope and + 90-diopter lens (Volk, USA). The status of DR in each eye was determined using the International Clinical Diabetic Retinopathy and Diabetic Macular Edema Disease Severity scales [25].

### Statistical Analysis

We used a power analysis and sample size software (PASS) to calculate the sample size. A sample size of 334 achieves 90% power to detect a clinically meaningful difference of 0.08, with a known standard deviation of 0.506 and significance level of 0.05. The calculation was further confirmed by placing the aforementioned values in the following formula, which yielded the same number of patients (334).

$$ n \, = \, \left( {\left( {\sigma d} \right)2 \, \left( {Z\beta + Z\alpha /2 \, } \right)2} \right)/{\text{difference2}} $$

where *n* is the sample size, *σ* is the standard deviation of the within-pair difference, difference is the clinically meaningful difference, *Zβ*   corresponds to power (1.28 = 90% power). *Zα*/2   corresponds to two-tailed significance level (1.96 for *α* = 0.05).

The data were analyzed using SPSS (version 26.0; IBM Corp., Armonk, NY, USA) and Microsoft Power BI 2021. Normality of the data was checked using the one-sample Kolmogorov–Smirnov test. Median (25th–75th quartiles) were reported for non-normally distributed quantitative variables. Categorical variables were reported as frequencies and percentages. A one-sample chi-square test was used to examine the difference in the observed frequencies of cataract location. The Mann–Whitney *U* test was used to compare various anthropometric and clinical variables in the presence and absence of cataracts. Pearson chi-square test was applied to identify the associations between anthropometric and clinical parameters among patients with diabetes with and without cataract. Post hoc tests were applied in cases of significant chi-square values using the Bonferroni adjustment. Binary logistic regression with backward conditional approach was applied to observe the log-odds between cataract (yes, no) and study variables. The ranking of the significant independent variables was performed using Wald statistics. Differences were considered statistically significant at α value of 0.05 (*p* < 0.05).

## Results

**Fig. 1:**
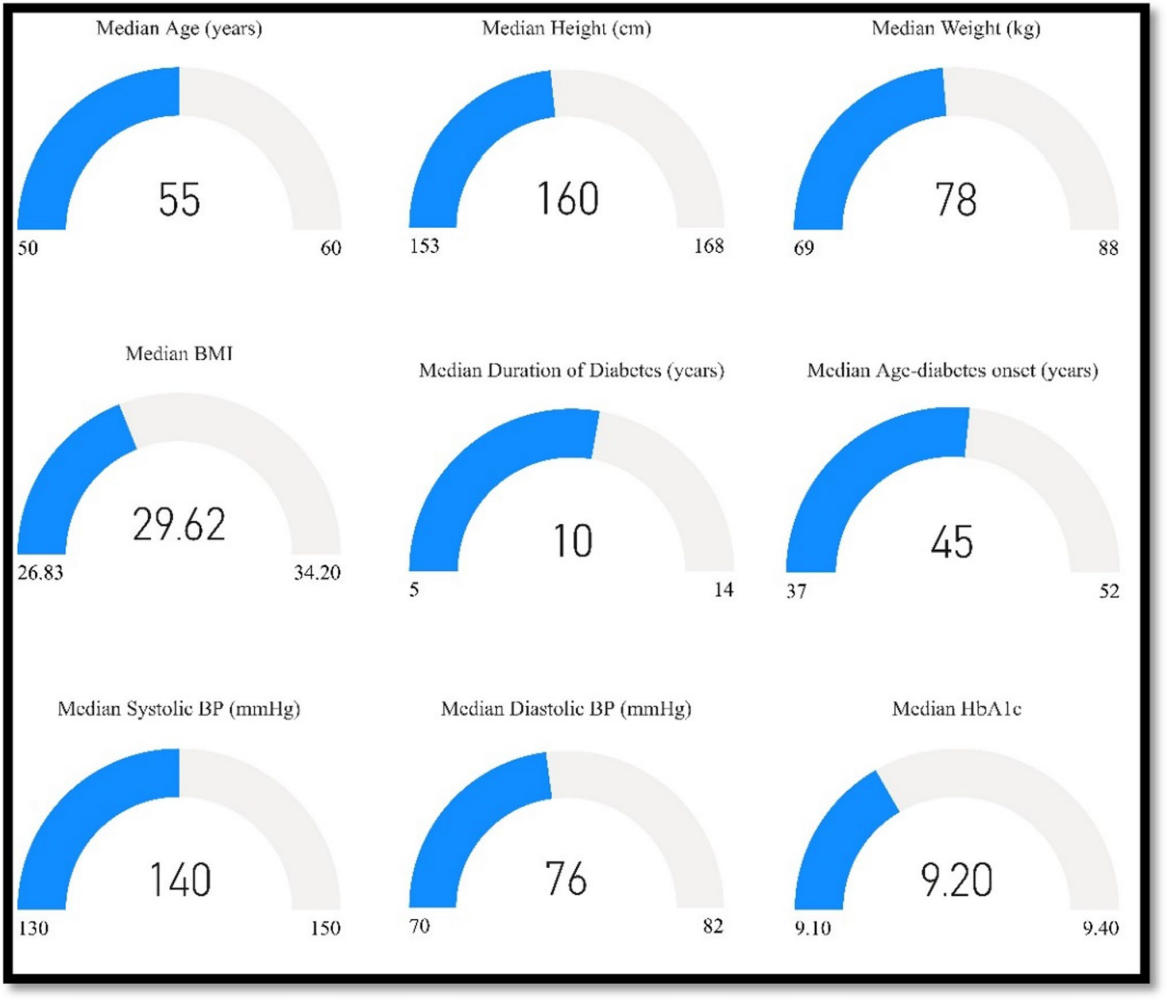
Median (25th–75th quartiles) for various study parameters

A total of 668 eyes of 334 patients with type 2 DM were examined. Cataract was present in 35.5% (*n* = 237) of the eyes; among them, 76 eyes (32%) were pseudophakic. The majority of the patients (595, 89.1%) were older than 40 years. Almost two-thirds of the patients were male and hypertensive, 424 (62.7%) and 419 (62.7%), respectively. Nearly half of the patients (328, 49.1%) were obese and approximately one-fourth of them (172, 25.6%) were on insulin. DR was present in almost one-third of the examined eyes (215, 32.2%) and most of the cases (194, 90.2%) were non-proliferative diabetic retinopathy (non-PDR). CSME was found in 89 eyes (13.3%). The detailed results are presented in Table 1. Anthropometric and clinical data variables were not normally distributed; the median (25th–75th quartile) value for the whole study sample is presented in Fig. 1.

**Table 1:** Socio-demographic, anthropometric and clinical characteristics of patients (*n* = 668)

Factor	*N*	%
Age		
≤ 40 years > 40 years	73 595	10.9 89.1
Gender		
Female Male	244 424	36.6 63.4
Hypertension		
No Yes	249 419	37.3 62.7
Medication		
Insulin OHG Not taking medication	172 470 26	25.8 70.4 3.8
Body mass index		
Normal Overweight Obese	92 248 328	13.8 37.1 49.1
Cataract^a^		
No Yes	431 237	64.5 35.5
Sub-type of cataract		
Cortical Posterior sub-capsular cataracts Nuclear sclerosis Mixed	73 49 14 25	45.3 30.5 8.7 15.5
Diabetic retinopathy (DR)		
No Yes	453 215	67.8 32.2
Stage of diabetic retinopathy (DR)		
Non-proliferative diabetic retinopathy Proliferative diabetic retinopathy (PDR)	194 21	90.2 9.8
Stage of non-proliferative diabetic retinopathy		
Mild Moderate Severe	104 73 17	53.6 37.6 8.8
Clinically significant macular edema		
No Yes	579 89	86.7 13.3

^a^Including pseudophakic lenses: 76 (32%)

From our sample, those with cataract (*n* = 237) were significantly older (*p* < 0.001), had lower weight (*p* = 0.022), had a longer duration of diabetes (*p* < 0.001), had a higher age of onset of diabetes (*p* < 0.001), and had higher systolic BP (*p* < 0.001) than those without cataract (*n* = 431). However, no significant difference was observed in the height (*p* = 0.352), BMI (*p* = 0.067), diastolic BP (*p* = 0.883) or HbA1c level (*p* = 0.112) between the two groups (Table 2).

**Table 2:** Comparison of anthropometric and clinical parameters among patients with and without cataract

Parameters	Cataract No Median (25th–75th) quartile *n*= 431	Cataract Yes Median (25th–75th) quartile *n*= 237	*p* value
Age	52.50 (46–59)	60 (55–66)	< 0.001*
Height	160 (153–168)	162 (154–168)	0.352
Weight	80 (70–89)	76 (67–87.25)	0.022*
BMI	30.09 (26.98–34.92)	29.05 (26.02–32.88)	0.067
Duration of diabetes	8 (4–15)	14 (6–20)	< 0.001*
Age onset of diabetes	43 (35–49)	48 (40–55.25)	< 0.001*
Systolic blood pressure	138 (130–150)	140 (130–160)	< 0.001*
Diastolic blood pressure	75 (70–80.75)	77 (70–83)	0.883
HbA1c	9.20 (9.20–9.40)	5.60 (5.20–6.37)	0.112

*Statistically significant at 5% level of significance

*BMI* body mass index, *HbA1c* hemoglobin A1c

DR and cataract were significantly associated (*p* < 0.001), and post hoc tests showed that DR was significantly present in patients with cataracts (106, 49.4%; *p* < 0.001). Moreover, a significant association was observed between age and cataract (*p* < 0.001), and the Bonferroni-adjusted post hoc test showed that cataract developed significantly more in patients with diabetes aged > 40 years (225, 37.8%; *p* < 0.001). Patient sex and cataract were also significantly associated (*p* = 0.012). Post hoc tests revealed that cataract incidence was significantly higher in males (164, 38.7%) than in females (71, 29.1%; *p* = 0.027). Hypertension and cataract were significantly associated (*p* = 0.001), and the post hoc test showed that the majority of the patients with cataracts were hypertensives (166, 39.6%; *p* < 0.001). However, no significant association was observed between cataract and BMI category (*p* = 0.302), type of medication (*p* = 0.509), stages of DR (*p* = 0.102), stages of non-PDR (*p* = 0.194), or CSME (*p* = 0.915; Table 3). Results of the one-sample chi-square test showed that most of the cataracts were cortical (73, 45.3%), followed by posterior sub-capsular cataract (PSC) (49, 30.5%), mixed (25, 15.5%) and nuclear sclerosis (NS) (14, 8.7%; *p* < 0.001).

**Table 3:** Association of cataract with gender, BMI, and clinical parameters

Factor	Cataract			*p *value
	No *n* (%)	Yes *n* (%)	Total	
Age				
< 40 years > 40 years	63 (86.3) 370 (62.2)	10 (13.7) 225 (37.8)	73 595	<0.001*
Gender				
Female Male	173 (70.9) 260 (61.3)	71 (29.1) 164 (38.7)	244 424	0.012*
Body mass index category				
Normal Obese Overweight	56 (61.4) 222 (67.7) 155 (62.5)	36 (38.6) 106 (32.3) 93 (37.5)	92 328 248	0.302
Medication				
Insulin Oral hypoglycemic (OHG) Not taking medication	112 (65.1) 305 (64.8) 14 (53.8)	60 (34.9) 165 (35.2) 12 (46.2)	172 470 26	0.509
Hypertension				
No Yes	180 (72.3) 253 (60.4)	69 (27.7) 166 (39.6)	249 419	0.001*
Diabetic retinopathy (DR)				
No Yes	324 (71.5) 109 (50.6)	129 (28.5) 106 (49.4)	453 215	<0.001*
Stages of diabetic retinopathy				
Non-PDR PDR	101 (52.1) 7 (33.3)	93 (47.9) 14 (66.7)	194 21	0.102
Stage of non-PDR				
Mild Moderate Severe	49 (47.1) 38 (52.1) 12 (70.6)	55 (52.9) 35 (47.9) 5 (29.4)	104 73 17	0.194
CSME				
No Yes	374 (64.6) 58 (65.1)	205 (35.4) 31 (34.9)	579 89	0.915

*Statistically significant at 5% level of significance;

*Non-PDR* non-proliferative diabetic retinopathy, *PDR* proliferative diabetic retinopathy,* CSME* clinically significant macular edema

Two binary logistic regression models were used separately. In model 1 (unadjusted), the dependent variable was cataract (yes, no) and the independent variables were DR, age, sex, and hypertension, which were chosen based on their significance from Table 3. Results showed that patients with DR were 2.464 times more likely to develop cataracts [odds ratio = 2.464; 95% confidence interval (CI) = –1.752 to 3.465, *p* < 0.001], and diabetics aged > 40 years were 4.398 times more likely to develop cataracts than those aged ≤ 40 years (odds ratio = 4.398; 95% CI = 2.061–9.382, *p* < 0.001]. In males, the chance of developing cataract significantly increased by 1.525 times [odds ratio = 1.525; 95% CI = 1.089–2.137, *p* = 0.014). Similarly, in hypertensive subjects, the chance of developing cataract significantly increased 1.710 times [odds ratio = 1.710; 95% CI = 1.212–2.412, *p* = 0.002; Table 4).

**Table 4:** Binary logistic regression (model 1) with backward elimination approach showing the relation between cataract and study variables

Parameters	*β*	S.E.	Wald	*p *value	OR	95% C.I.		
Diabetic retinopathy	0.902	0.174	26.89	<0.001*	2.464	1.752		3.465
Age	1.481	0.387	14.674	< 0.001*	4.398	2.061		9.382
Hypertension	0.536	0.175	9.344	0.002*	1.710	1.212		2.412
Gender	0.422	0.172	6.022	0.014*	1.525	1.089	2.137	

*Statistically significant at 5% level of significance

*S.E*. standard error, *OR* odds ratio, *C.I.* confidence interval

In logistic regression model 2, the dependent variable was the presence and absence of cataract, whereas the independent variables were adjusted for age, sex, BMI, systolic BP, diastolic BP, duration of diabetes, HbA1c, and DR). The model chi-square value was significant at the 1% level of significance, confirming the appropriateness of the fitted model. The classification accuracy achieved using the fitted model was 76.9%. The results showed that diabetics aged > 40 years were 3.089 times more likely to develop cataracts than those aged ≤ 40 years (adjusted odds ratio = 3.089; 95% CI = 1.401–6.814, *p* = 0.005). Similarly, patients with DR were 1.668 times more likely to develop cataracts (OR = 1.668; 95% CI = 1.135–2.451, *p* = 0.009). As the duration of diabetes increased by 1 year, the chance of developing cataract significantly increased by 1.049 times (adjusted odds ratio = 1.049; 95% CI = 1.024–1.074, *p* < 0.001). Moreover, with an increase of 1 mmHg in systolic BP, the chance of developing cataract significantly increased by 1.010 times (adjusted odds ratio = 1.010; 95% CI = 1.002–1.215, *p* = 0.039). However, sex (*p* = 0.390), BMI (*p* = 0.460), HbA1c (*p* = 0.150), and diastolic BP (*p* = 0.857) were not significant, as predicted by the model (Table 5).

**Table 5:** Binary logistic regression (model 2) with backward elimination approach showing the relation to cataract, adjusted for study variables

Parameters	*β*	S.E.	Wald	*p* value	Adjusted OR	95% C.I.	
Age	1.128	0.404	7.811	0.005*	3.089	1.401	6.814
Gender	0.163	0.190	0.740	0.390	1.177	0.812	1.707
BMI	−0.010	0.013	0.547	0.460	0.991	0.966	1.016
Systolic BP	0.010	0.005	4.253	0.039*	1.010	1.002	1.215
Diastolic BP	−0.002	0.009	0.032	0.857	0.998	0.981	1.016
Duration of diabetes	0.048	0.012	15.482	< 0.001*	1.049	1.024	1.074
HbA1c	−0.111	0.077	2.073	0.150	0.895	0.769	1.041
DR	0.511	0.196	6.773	0.009*	1.668	1.135	2.451

^*^Statistically significant at 5% level of significance

*BP* blood pressure, *BMI* body mass index, *DR* diabetic retinopathy, *S.E.* standard error,* O*R odds ratio, *C.I*. confidence interval.

## Discussion

To the best of our knowledge, this is the first study in the Kingdom of Saudi Arabia that investigated the prevalence of cataract among the diabetic population; currently, there are limited population-based studies comparing DR patients with non-DR patients with regard to cataract development.

The prevalence of cataracts in our sample was 35.5%, which is within the reported prevalence in the literature (18–50%) [26]. Although many studies worldwide have shown that diabetes is associated with an increased risk for developing cataracts, the associated factors that increase or decrease this risk remain unclear [11, 13, 16–19, 22, 27, 28]. In this study, we report on some of these factors. Exploring the relationship of DM and cataract subtypes further, we found that cortical cataract was the most frequently observed subtype, followed by PSC, while NS was minimal, which is consistent with most of the previous studies [13, 17, 19, 21, 29, 30]. Two studies reported contrasting finding—that is, NS was identified as the predominant subtype [26, 31]. Srinivasan et al. attributed the higher incidence of nuclear cataract in their study to a warmer climate and more exposure to the sun, relative to other studies; however, in our region, the climate is even hotter and sun exposure is much greater.

DR and cataract were significantly associated in our study; both unadjusted (odds ratio = 2.464; 95% CI = 1.752–3.465, *p* < 0.001) and adjusted (odds ratio = 1.668; 95% CI = 1.135–2.451, *p* = 0.009) models were significant (*p* < 0.001), respectively. This is in accordance with the two studies conducted in the United Kingdom [32, 33]. A study in Brazil with a similar sample size to ours reported that cataract occurs four times more in diabetics with DR, which is 2.5 times what we found (1.67 times). This result may be due to the fact that they did not control for the duration of diabetes, which is a major confounding factor. Most of the risks of cataract in DR patients are due to the longer duration of DM, as DR is usually a late complication [34]. In the Wisconsin study, DR had the strongest association with cataract, but the study investigated type 1 DM [18]; this study also reported that the severity of DR is one of the predictors of cataract surgery. In our study, there was no increase in the association of cataracts with any DR stage.

The incidence of cataract in diabetics with macular edema was found to be higher than that in the general diabetic population in the UK [32]. We did not find a significant relationship between cataract and CME. DR is mainly a vascular complication, while cataract is thought to be a biochemical complication of high blood glucose levels [13, 15]. Why DR is a risk factor for cataract development needs to be studied further, as it may unveil some cataract pathology.

Our results showed a statistically significant association of cataract in patients with type 2 DM with age and duration of DM, and it seems that there is almost consensus on these two factors in the literature [15, 18, 19, 21, 26, 28, 31–35]. Some studies reported higher risk for diabetic cataract in the younger age groups, which we did not find [11, 32, 36, 37].

Many previous studies have reported that diabetic women are at a higher risk of cataracts than diabetic men [21, 28, 31–33, 35, 36]; nevertheless, the underlying reason for this observation is unclear. However, theories have been suggested to explain that, like the role of estrogen in protecting the lens from oxidative stress, its decrease after menopause would increase the risk for cataract, as well as albumin to total protein and serum triglyceride levels in women^.^ [31, 35]. In our study, logistic regression model 1 revealed a significant association between sex and cataract (*p* = 0.012). The post hoc test revealed that cataract was significantly higher in men than in women (*p* = 0.027), while in logistic regression model 2, no significant association was predicted. A possible reason for this result may be the higher number of males than females in the study sample, and the lower exposure of females to the sun in Saudi Arabia; however, further studies are required to confirm this speculation. Other studies have also found no significant difference in cataract development between sexes [38].

We found a significant association between hypertension and cataracts, specifically systolic BP. This finding is consistent with previous studies [18, 27, 34]. Similar to previous studies, we did not find a significant association between cataract and either BMI or obesity [31, 34]. However, other studies have related BMI to the incidence of cataract [27, 39].

In the present study, we found no association between HbA1c and cataract; a similar finding was reported by Esteves et al. in Brazil [34]. They attributed this result to their study design or to the good metabolic control in their sample. However, many other researchers have reported a significant association between cataract and HbA1c [11, 32, 33, 37]. One study reported a significant association between HbA1c and posterior sub-capsular cataracts, but not nuclear cataracts [21].

The incidence rates of cataract in our study were not affected by the use of insulin, whereas some previous studies found increased risk in patients using insulin [18, 32, 33]. This increased risk may be due to the higher percentage of diabetic complications in insulin users.

We examined a new factor, the age of onset of DM, and its relation to the development of cataract. Patients with diabetes with cataract had a significantly higher age of onset of diabetes (*p* < 0.001). To the best of our knowledge, this finding has not been previously reported. The possible reason for this association is that as DM develops late in life, patients are already at a higher risk of senile cataract. However, further exploration is needed to ascertain whether it is an independent variable.

Our study has several strengths. The source of our sample was a well-organized and updated primary care database and the sample was randomly selected from that source with very high response rate. The information on lens and fundus status was collected through the most accurate and reliable way (slit-lamp and indirect ophthalmoscope). We included several potential confounders in the analysis, like BP, BMI, insulin use, Hba1cand age of onset of diabetes.

The weakness in our study could be due to depending on history by patient in regard of insulin use and duration and age of onset of diabetes. In addition, BP was taken once in the clinic increasing the possibility of falls positive readings.

## Conclusion

DR is an independent risk factor for cataract development in patients with type 2 DM. Other risk factors included age, duration of DM, and hypertension. Sex, BMI, HbA1c, and insulin use still need to be explored further. Age at onset of DM is a new factor that has been found to be significantly associated with the development of cataracts. Future studies should further investigate the association of age of onset in DM and cataract development.
